# Neural stem cells respond to stress hormones: distinguishing beneficial from detrimental stress

**DOI:** 10.3389/fphys.2015.00077

**Published:** 2015-03-11

**Authors:** Yassemi Koutmani, Katia P. Karalis

**Affiliations:** ^1^Center for Experimental Surgery, Clinical and Translational Research, Biomedical Research Foundation of the Academy of AthensAthens, Greece; ^2^Endocrine Division, Children's Hospital, Harvard Medical SchoolBoston, MA, USA

**Keywords:** neural stem cells, stress, stress hormones, glucocorticoid, adult neurogenesis, nervous system development

## Abstract

Neural stem cells (NSCs), the progenitors of the nervous system, control distinct, position-specific functions and are critically involved in the maintenance of homeostasis in the brain. The responses of these cells to various stressful stimuli are shaped by genetic, epigenetic, and environmental factors via mechanisms that are age and developmental stage-dependent and still remain, to a great extent, elusive. Increasing evidence advocates for the beneficial impact of the stress response in various settings, complementing the extensive number of studies on the detrimental effects of stress, particularly in the developing brain. In this review, we discuss suggested mechanisms mediating both the beneficial and detrimental effects of stressors on NSC activity across the lifespan. We focus on the specific effects of secreted factors and we propose NSCs as a “sensor,” capable of distinguishing among the different stressors and adapting its functions accordingly. All the above suggest the intriguing hypothesis that NSCs are an important part of the adaptive response to stressors via direct and indirect, specific mechanisms.

## Introduction

Embryonic stem (ES) cells are characterized by their unique ability for self-renewal and their potential to differentiate into any type of functional somatic cell. During development this potential is progressively diminished as ES cells become lineage-committed precursors. Thus, in the central nervous system neural stem cells (NSCs) are considered the lineage precursors of all neuronal and glial cells (Weiss et al., 1996; Gage, 2000; Kriegstein and Alvarez-Buylla, 2009). Generation of functional neurons by NSCs is an elegant, dynamically regulated process, extremely active during gestation, reduced in the early postnatal period, and maintained in low rates in the adult. During the embryonic period new neurons arise from the ventricular zone and migrate to different regions ultimately populating the entire brain (Altman and Bayer, 1990a,b). In the adult, NSCs reside in specific neurogenic “niches,” more specifically the subventricular zone (SVZ) of the lateral ventricles, the subgranular zone (SGZ) of the hippocampal dentate gyrus (DG), and several other brain regions recently identified (Ihrie and Alvarez-Buylla, 2011; Decimo et al., 2012; Gage and Temple, 2013; Ernst et al., 2014). As anticipated by the enormous importance of neurogenesis, this process is under the strict control of a multitude of intrinsic and extrinsic factors. Intrinsic factors are mainly transcription factors regulated by signaling pathways driven by Notch, Ephrin-B, neurotrophin receptors and others (Altman and Das, 1967; Kaltezioti et al., 2010; Remboutsika et al., 2011; Decimo et al., 2012). One other important parameter is the epigenetic status of stem cells enabling them to sense and respond to the complex net of extrinsic signals presented in the “niche.” Despite the necessity for a stable, genetically determined mechanism regulating the production of new neurons, neurogenesis is a plastic process controlled by the environment (Cameron and Gould, 1994; Blaschke et al., 1996; Tanapat et al., 1999, 2005; Karishma and Herbert, 2002; Baud et al., 2005). One of the most complex physiological processes with prominent effects in both the embryonic and the adult NSCs, is stress.

Stress response is the physiologically raised adaptation of an organism to any challenge of its homeostasis. Under non-stress conditions, stem cell “niches” represent a unique microenvironment where interactions between stem cells, other resident cells and soluble autocrine, paracrine, and endocrine signals ensure the optimal system function. Stressors modify this microenvironment, whereas NSCs are not spared by the systemic stress responses driving adaptation. Hypoxia, inflammation, metabolic or psychological stressors have been shown to provoke the altered NSCs “behavior” as a reaction to the modified environment. In mammals systemic stress response is driven by the orchestrated activation of the hypothalamic-pituitary-adrenal (HPA) axis and the catecholaminergic system (Bishop and King, 1999). The necessary step for the initiation of the stress response is the secretion of the neuropeptide corticotropin-releasing hormone or factor (CRH or CRF) that ultimately drives the release of adrenal glucocorticoid (Chen et al., 2004). Glucocorticoid (cortisol in humans and corticosterone in rodents) is the end product of the HPA axis exerting a negative feedback in the brain in order to control for glucocorticoid overexposure.

Despite the widely recognized impact of stress hormones on neurogenesis, little progress has been made in the elucidation of the molecular mechanisms that underlie this outcome. The current review examines the existing knowledge on the effects of the stress hormones in the biology of NSCs, and introduces the NSCs cellular machinery as a sensor capable of distinguishing between the beneficial and detrimental stress. Determining the molecular components of the actions of stress hormones on NSCs activity will be a hallmark in the research on stress but also in the field of regenerative medicine in general.

## Factors that influence NSCs responsiveness to stress hormones

**Figure 1:**
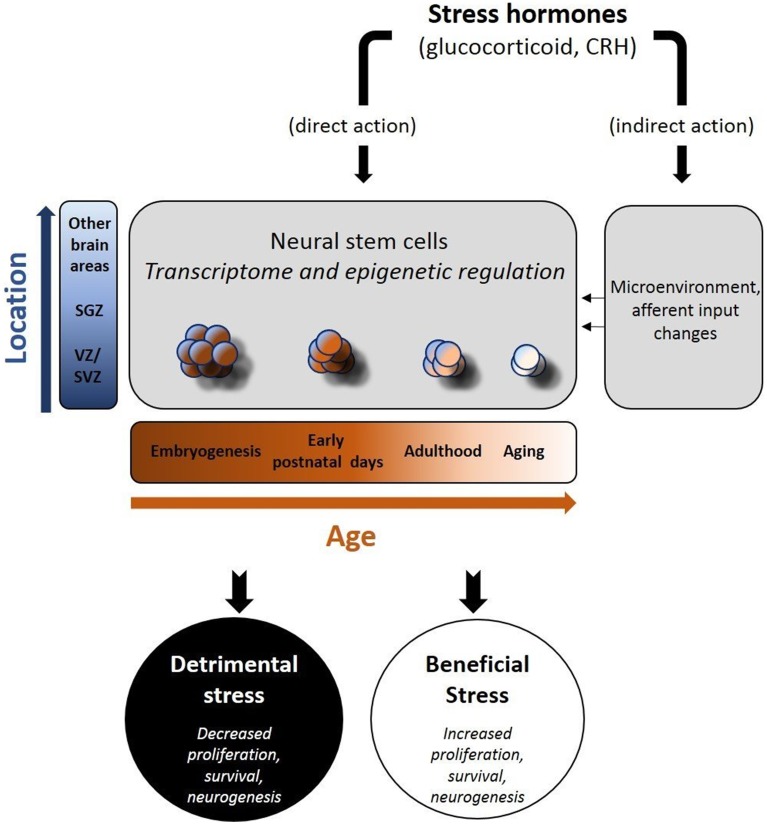
**Schematic depiction of the effects of stress hormones on neural stem cells (NSCs)**. Stress hormones may exert beneficial or adverse effects on neural stem cell activity (proliferation, survival, differentiation to mature neurons) via their direct and indirect actions. As the transcriptome and the epigenetic profile of NSCs change (in a time and space-dependent manner), NSCs react either due to direct exposure to stress hormones (direct action) and/or following the stress hormone-induced changes in their “niches(s)” (indirect action). The nature of the stressor, the location of NSCs and the time in the development are major determinants of the short- and long-term effects in brain function.

Stress hormones act on NSCs during development but also in adult life, via distinct and, in several cases, opposing ways. A striking difference between embryonic and adult NSCs is that in the prenatal or early-postnatal period, stress has a lasting impact on their “behavior” with some of its effects recognizable in adult life or even during aging (Bose et al., 2010; Androutsellis-Theotokis et al., 2013; Belnoue et al., 2013; Peffer et al., 2014; Provencal and Binder, 2014; Urban and Guillemot, 2014; Ortega-Martinez, 2015). In contrast, stress-induced changes in the adult neurogenic populations, are mostly reversible (McEwen, 1999; McEwen and Magarinos, 2001; Duman, 2002; Morais et al., 2014). The exact reasons for the above differences are not clear, but increasing evidence suggests that epigenetic regulation may be a major contributor for stress effects during development (Figure 1). Furthermore, the strict control of the embryonic NSCs to guarantee the uneventful developmental programing, suggests that any threatening homeostatic perturbation has the potential to impinge on the function of specific brain structures. Many models of early life adversity have been developed in rodents, in order to study the impact of stress hormones in neurogenesis (Pryce et al., 2005). The most commonly applied prenatal stress models include physical stressing of the pregnant mother or administration of glucocorticoid receptor (GR) ligands e.g., dexamethasone (DEX), to simulate the activation of the HPA axis (Welberg and Seckl, 2001). Similarly, the most widely applied early postnatal stress models have mainly concentrated on the psychological stress induced by maternal deprivation (Zhang et al., 2013). Notably, during early postnatal period, stress has been shown to exert positive effects on neurogenesis, in contrast to the long-lasting effects recognizable in adults, raising the hypothesis for strong association between early life stress and neurodegeneration (Oomen et al., 2009; Suri et al., 2013). In the adult brain it seems that NSC responsiveness to stress is modified by aging, in part explained by the age-dependent decrease in the expression of GRs (Seki and Arai, 1995; Kuhn et al., 1996; Garcia et al., 2004; Simon et al., 2005; Leuner et al., 2007; Abdanipour et al., 2015).

The specialized “niche” environment is another crucial factor in the regulation of NSC physiology. A recent report based on comparative transcriptomic analysis between mouse SGZ and SVZ cells shows site-specific differences in the regulatory networks of locally expressed transcription factors (Ertaylan et al., 2014). An extensive number of brain region-specific factors, such as epidermal growth factor (EGF), fibroblast growth factor (FGF), Sonic Hedgehog and Wnt signaling, act on NSC populations and modify their transcriptome profile, with direct impact on their responses to stress hormones (Ikeya et al., 1997; Kalyani et al., 1997; Gritti et al., 1999; Raballo et al., 2000). As example of the interplay between the locally expressed transcription factors and stress hormones, is the suppressive effects of glucocorticoid on Sonic Hedgehog-induced proliferation in mouse NSCs and the Wnt signaling antagonist DKK1-mediated inhibition of proliferation and neuronal differentiation, induced by DEX in human embryonic NSCs (Heine and Rowitch, 2009; Moors et al., 2012).

The most extensively studied stress-responsive neurogenic area has been the SGZ, located within the DG of the hippocampus. This is an area with identified strong neurogenic potential also in humans, where it is functionally associated with very important processes such as cognition, emotion, and pattern separation (Eriksson et al., 1998; Leutgeb et al., 2007; Curtis et al., 2011; Nalloor et al., 2012; Rubin et al., 2014). Briefly, neuronal precursor cells derived from the SGZ migrate radially to the upper granular layers, differentiate into mature neurons and progressively integrate in the local networks (Esposito et al., 2005; Ming and Song, 2005; Faigle and Song, 2013). There is strong evidence that exposure to glucocorticoid results in dramatic reduction of neuronal precursors in the SGZ (Cameron and Gould, 1994; Wong and Herbert, 2006; Brummelte and Galea, 2010). Studies in rodents have shown that glucocorticoid targets also the extra-hippocampal cells proliferation, although in a different manner. Remarkably, NSCs that are hosted in the rat SVZ do not show a dramatic response, such as the SGZ, to chronic treatment with corticosterone (Alonso, 2000).

Recently, a number of studies in different species identified distinct NSC “niches” in the adult hypothalamus, the cerebral cortex, the cerebellum, the olfactory bulb, the retina, and the striatum (Mackay-Sim and Kittel, 1991; Tropepe et al., 2000; Carter et al., 2004; Kokoeva et al., 2005, 2007; Ponti et al., 2006, 2008; Leung et al., 2007; Decimo et al., 2012; Ernst et al., 2014). As of now, the effects of stress hormones in these cell populations remain unknown although a variety of brain functions controlled by these areas are regulated by glucocorticoid.

Gender differences in the stress response have been well-documented in several species. It seems that basal circulating glucocorticoid levels are higher in females, a difference normalized by aging (Falconer and Galea, 2003; Westenbroek et al., 2004; Zuena et al., 2008). Interestingly, the expression of steroid receptors in undifferentiated NSCs display sexual dimorphism as well, providing a possible explanation for their differences in response to glucocorticoid (Waldron et al., 2010; Loi et al., 2014).

## Negative and positive effects of stress hormones on NSCs

### Negative regulation

In the majority of studies, exposure to stressors has been associated with inhibition of neurogenesis both in fetal and adult life. Constant or interrupted exposure of rodent embryonic NSCs to the synthetic glucocorticoid DEX compromises their proliferation and survival (Bose et al., 2010; Samarasinghe et al., 2011). Recently, the responsiveness of embryonic NSCs to glucocorticoid was linked to the direct, acute genomic effects of the activated GRs, known as transcriptional regulators (Androutsellis-Theotokis et al., 2013; Peffer et al., 2014). Moreover, *in vitro* exposure of rat ES cells to DEX induced heritable alterations and changes in the expression of genes associated with cellular senescence and proliferation (Sippel et al., 2009; Bose et al., 2010). Notably, the expression pattern of GRs in mouse embryonic NSCs changes with differentiation, *in vivo* or *in vitro*. Furthermore, expression of GRs is region-specific, adding another variable in the responsiveness of NSCs to stressors (Androutsellis-Theotokis et al., 2013; Tsiarli et al., 2013).

During early postnatal development, the neurogenic pools for the whole extra-uterine life are committed in the restricted areas. Exogenous administration of DEX during that period led to shrinkage of NSC pool in the adult hippocampus. The latter was correlated with compromised learning and memory (Ortega-Martinez, 2015; Ortega-Martinez and Trejo, 2015). This data suggests that NSCs can be harmed by pharmacological doses of glucocorticoid and exposure of neonates to this drug should be done with caution.

During adulthood, acute and chronic stressors can activate the HPA axis, resulting in elevated glucocorticoid levels and reduced neurogenic activity (Gould et al., 1997, 1998, 1999; Lagace et al., 2010). In rodents, paradigms of acute psychological stress such as exposure to the odor of natural predators, have been associated with decreased cell proliferation and differentiation of the immature neurons in the DG of hippocampus (Tanapat et al., 2001; Mirescu et al., 2004; Hill et al., 2006; Kambo and Galea, 2006). Similar results have been demonstrated in mice exposed to social defeat or following foot- or tail-electric shock (Duman, 2002; Malberg and Duman, 2003; Yap et al., 2006; Fornal et al., 2007; Lagace et al., 2010). Chronic stress paradigms like chronic social stress in rodents and primates resulted in significant reduction in NSC proliferation in the DG (Czeh et al., 2001; Simon et al., 2005; Perera et al., 2007; Ferragud et al., 2010). Noise-induced stress, restrain stress, or the chronic use of multiple mild stressors also decreased NSC proliferation, although the main effect was the compromised survival of newly-born neurons (Pham et al., 2003; Lee et al., 2006; Oomen et al., 2007; Gonzalez-Perez et al., 2011).

In line with the above, exogenous administration of corticosterone led to reduced number of proliferating cells and survival of NSCs in the adult DG. Furthermore, glucocorticoid deprivation following adrenalectomy, stimulated neurogenesis (Gould et al., 1992; Cameron and Gould, 1994; Wong and Herbert, 2006; Brummelte and Galea, 2010). Recent data suggests that challenge with glucocorticoid may impact on the differentiation of NSCs in the hippocampus. Thus, DEX-treated adult NSCs showed impaired differentiation toward the neuronal phenotype, whereas corticosterone-treated mouse hippocampal NSCs were driven toward oligodendrogenesis at the expenses of neurogenesis (Heberden et al., 2013; Chetty et al., 2014). Similar effects were observed in the spinal cord, where treatment with high-dose of corticosterone for spinal cord injury, reduced NSCs proliferation locally (Schroter et al., 2009). Finally, *in vitro* exposure of murine NSCs to corticosterone triggered both cell death and proliferation in a concentration-dependent manner (Wolf et al., 2009; Abdanipour et al., 2015).

Remarkably, the majority of studies used exogenous administration of glucocorticoid whereas *in vivo* this is a very tightly self-regulated system, with the exception of limited cases such as tumors or following uncontrolled exposure to severe stressors. Thus, in addition to the GR-mediated effects, indirect actions of stress hormones should be considered, particularly given the relatively low abundance of GRs in NSCs compared to the mature neurons (Cameron et al., 1993; Garcia et al., 2004). Recent studies, during *in vitro* and *in vivo* differentiation of mouse embryonic NSCs revealed brain region-specific differences in the expression pattern of GRs (Androutsellis-Theotokis et al., 2013; Tsiarli et al., 2013). Finally, glucocorticoid may also affect neighboring neuronal or non-neuronal cells driving them to apoptosis or modifying their functions such as their inputs to local NSCs pools. Along these lines, cytokines released by activated microglia may have toxic effects on neuronal precursors, regulating indirectly their activity (Ekdahl, 2012).

### Positive regulation

Surprisingly, although HPA axis activation has been strongly associated with suppression of neurogenesis, there are some stressors that consistently increase the proliferation rate and enhance the survival of NSCs. For example, running and physical exercise, both strong activators of the HPA axis and thus increasing circulating glucocorticoid levels, they induce proliferation and survival of newborn neurons (van Praag et al., 1999, 2002; Droste et al., 2003; Makatsori et al., 2003; Stranahan et al., 2006; Snyder et al., 2009; Yi et al., 2009; Schoenfeld and Gould, 2012; Saaltink and Vreugdenhil, 2014). Similarly, positive psychological challenge such as housing in an enriched environment, increases the circulating glucocorticoid levels and supports survival of newborn neurons and protection of NSCs from the adverse effects of aging (van Praag et al., 1999; Kempermann et al., 2002). Sexual experience and learning have been also associated with increased circulating glucocorticoid levels and induction of the neurogenic activity (Bonilla-Jaime et al., 2006; Leuner et al., 2010). All the above “stressful” experiences allow not only for protection of NSCs from the negative effects of glucocorticoid but even more, they exert positive effects on NSCs. A common characteristic of the above stressors is that they have a strong “rewarding” component, associated with the release of neuropeptides/neuromodulators, such as endogenous opioids, dopamine, or neurotrophins such as the brain-derived neurotrophic factor (BDNF). All these neuromodulating peptides seem to protect NSCs from the toxic effects of glucocorticoid and, -most likely, to promote neurogenesis (Persson et al., 2004; Sairanen et al., 2005; Ying et al., 2005; Winner et al., 2009; Taliaz et al., 2010).

Although the precise mechanisms mediating these beneficial effects of particular stressors to NSCs remain unknown, there is data suggesting implication of other cell types, neighboring the stem cells, such as microglia and the astrocytes. Activation of additional steroid receptors such as progesterone and estrogen that may modulate the glucocorticoid effects has been suggested. Moreover, stress hormones may act on the granule cell afferents that also express GRs. For example, it has been shown that manipulation of the cholinergic inputs or blockade of NMDA receptors, glutamate receptors or serotonin receptors (5-HT1A) that supply synaptic signals to DG cells from other brain regions such as the entorhinal cortex, influence adult neurogenesis in the SGZ “niche” (Meijer and de Kloet, 1994; Cameron et al., 1995; Flugge et al., 1998; Kotani et al., 2006; Zhao et al., 2008; Frechette et al., 2009; Maekawa et al., 2009). In support of the above and of direct translational significance is the observation that GRs are required to mediate the neurogenic effects of the antidepressant serotonin reuptake inhibitor sertraline (Anacker et al., 2011).

In contrast to the great number of studies looking into the effects of glucocorticoid in NSCs, there is limited information on the effects of CRH, the neuropeptide required for the induction of the stress response, in this process. Although CRH is a positive regulator of glucocorticoid release, its effects in several cases is been in opposite directions. For example, CRH has been recently shown to protect neurons from the damaging effects of hypoxia (Valadas et al., 2012). According to our working hypothesis described above, this effect of CRH is in line with its homeostatic actions in challenging conditions. Studies with the *Crh*-null mice show that their inability to raise an adequate stress response is not to their overall benefit, in accordance with the first described by Hans Selye beneficial effects of the adaptive response (Selye, 1975). We have recently reported that CRH induces proliferation of embryonic NSCs via direct CRH receptor-mediated effects and protects from apoptosis *in vitro* and *in vivo*. Most importantly, CRH can oppose the glucocorticoid-mediated toxic effects on NSCs, revealing the complexity of the stress response in neurogenesis (Koutmani et al., 2013). These observations highlight the dual action of the stress hormones on the activity of NSCs that enables them to act as wide-spectrum neuromodulators.

## Perspectives

A major scientific challenge of our times is to successfully implement advances in stem cell biology for the treatment of human diseases. Although ES cells have the capacity to give rise to all cell lineages, their therapeutic potential is limited due to teratoma formation and ethical concerns (Blum and Benvenisty, 2008, 2009). Induced pluripotent stem cells (iPS cells), bone marrow mesenchymal stem cells, and dental pulp stem cells, able to differentiate to neuronal lineages both *in vitro* and *in vivo* after transplantation, have been used to repair injured neurons. Unfortunately, so far they have only shown to result in modest recovery most likely due to failure to compensate for the associated loss of yet unidentified factors of the micro-environment (Jiang et al., 2002; Jin et al., 2002; Imitola et al., 2006; Yiu and He, 2006; Charil and Filippi, 2007; Tetzlaff et al., 2011; Mothe and Tator, 2012; Xiao and Tsutsui, 2013). These important obstacles in the transplantation-mediated CNS repair might be overcome by our better understanding of the endogenous NSC and “niche” biology and the leverage of this knowledge for therapeutic purposes.

The previous studies reviewed above suggest that stress hormones are critical regulators of NSC functions during development and in adult life, and support important regulatory mechanisms driving brain homeostasis. Impaired neurogenesis is tightly linked to many psychiatric diseases such as depression and post-traumatic stress disease, while it is also implicated in the pathogenesis of neurodegenerative disorders such as Alzheimer's and Parkinson's disease (de Kloet et al., 2005; Eisch and Petrik, 2012). A number of new drugs from the spectrum of disorders are designed to mimic or antagonize specific actions of the stress hormones (Fitzsimons et al., 2009). Elucidating the specific effects of stress hormones and most importantly, the molecular machinery implicated in NSC biology could provide unique insights in the treatment of diseases of the nervous system without raising ethical concerns.

### Conflict of interest statement

The authors declare that the research was conducted in the absence of any commercial or financial relationships that could be construed as a potential conflict of interest.
